# Determination of Methanol Concentrations in Traditional Herbal Waters of Different Brands in Iran

**Published:** 2011

**Authors:** Seyed Reza Mousavi, Mohssen Namaei-Ghassemi, Massomeh Layegh, Monavar AfzalAghaee, Manssoreh vafaee, Gholamali Zare, Toktam Moghiman, Mahdi Balali Mood

**Affiliations:** 1 *Medical Toxicology Research Center, Mashhad University of Medical Sciences, Mashhad, Iran*

**Keywords:** Concentration, Gas Chromatography, Herbal Water, Methanol

## Abstract

**Objective(s):**

Herbal waters are extensively used in most parts of including . Visiting a patient with total blindness due to daily ingestion of around 200 ml of herbal water (Plant forty water) per day for six months was the rational for methanol determination in all herbal waters available in markets.

**Materials and Methods:**

A total of two hundred and nineteen bottles of herbal waters were randomly bought from market. Methanol concentration was determined by gas chromatography, using a Flame Ionized Detector. Benzene (1000 mg/l) was applied as the internal standard. Collected data was analyzed by SPSS software (version 11.5), using appropriate descriptive statistical tests.

**Results:**

Forty six different herbal waters from three main producing factories (A, B and C) were tested. Highest methanol concentration was measured in dill water of A (1208±202.74 mg/l), concentrated rose water of A (1017.41±59.68 mg/l) and concentrated rose water of B (978.52±92.81 mg/l). Lowest methanol concentration was determined in *Trachyspermum copticum *water of B (18.93±1.04 mg/l), cinnamon and ginger water of B (29.64±10.88 mg/l) and rice skin water of A (41.33±7.85 mg/l). Mean methanol concentrations of herbal waters including ginger, cinnamon, dill, peppermint, alfalfa, and plant forty from A, B and C were 374.69, 209.81 and 280.12 mg/l, respectively (*P*< 0.001).

**Conclusion:**

Methanol concentration in all herbal waters, especially rose water of the three producers was very high that may induce toxicity in people taking these products regularly for a long time.

## Introduction

Herbal water (plant water, herbal essence, aromatic water, hydrola) is referred to the liquid obtained from the cooled steam (distillation) of medicinal plants. It can also be called aqueous herbal extract which is a water-based preparation of a plant containing the biologically active portion of the plant without its cellular residue. Different parts of plants, such as flowers, fruits, leaves, seeds and roots have long been used to produce herbal waters. Fermentation of the fibers (cellulose) of the plant may induce methanol or methyl alcohol. Methanol is the simplest type of alcohol that its oral use and even vapor exposure is extremely toxic for humans (1). 

Methanol enters the body through drinking, breathing and skin absorption; its long time usage without a protection device (mask and gloves) can be hazardous (2). Methanol is cheap and readily available in the markets worldwide. It has long been used in the production of imitated spirits and wine, causing many cases of blindness and death in China, Brazil, Elsalvador, India and Taiwan (3). Methanol's lethal dose is 100 to 120 ml and its toxic effects usually manifest a few hr after consumption. Most common clinical manifestations of acute methanol poisoning include headache, dizziness, nausea, imbalance, distress, drowsiness, blurred vision, metabolic acidosis and eventually delirium and death. Although low level exposure of methanol may be asymptomatic, frequent multiple exposure, particularly oral intake may induce toxicity (4).

Pectins occur as structural polysaccharides in the middle lamella and primary cell walls of higher plants (5). Pectin methylesterase (PME) de-esterifies pectins to low-methoxyl pectins, resulting in the formation of methanol (6).

In the manufacturing process of herbal medicine, methanol is usually used as a solvent to extract natural ingredients; the residual level of methanol in such products is a matter of great concern (7).

Detection of pectin methylesterase activity in the presence of methanol during grape storage may reduce methanol concentration (8).

It is important to note that the formation of methanol during fermentation in a herbal water is dependent on a number of factors such as type of the plant and its species, plant health, maceration method, fermentation temperature and pectolytic enzyme treatment. 

Herbal waters are used as dietary supplements and alternative medicine and are commonly used for flavoring in baking and other cooking, much like rose water. Therefore, its safety and toxic free compounds like methanol is very vital to consumers. Frequent consumptions of some herbal waters such as mint, rose water and plant forty water may induce severe toxicity, particularly blindness. 

The corresponding author visited a patient with total blindness with unknown origin. Through the investigation for a cause of the blindness, it was discovered that daily ingestion of around 200 ml of herbal water (Plant forty water) for six months may contain a toxic substance. Methanol concentration in the remaining herbal water was very high (380 mg/l). It was thus aimed to estimate concentration of methanol in a large number of herbal waters available in markets.

## Materials and Methods

In this study the largest factories producing herbal waters in Khorasan Razavi province (Iran) were primarily identified. The initial investigators (the corresponding and 2^nd^ authors) visited the largest producer of herbal waters in the province following coordination with the management board. The process of producing herbal waters in this factory was recorded and the summary is as follows:

When a specific herb or plant is brought to the factory, depending on its type being fresh or dry, it initially goes into the sorting process. If the plant is fresh, it is washed out and transferred to the steel distillation cauldron. For dried herbs, scrubbing or grinding may be needed before transferring to the cauldron. After adding water, the cauldron is heated and the vapor is passed through the distillation device. The final product is a colorless liquid which is transferred to a steel preservation storage. After filtration and pasteurization procedures in the packing saloon, the herbal water is packed and stored in a springhouse.

The herbal water products of this factory (A) and two other main producers (B) and (C) were randomly selected and purchased in summer 2008 in markets. All available different herbal waters of the factories A, B and C were 38, 21 and 14 types, respectively. Three samples of each herbal water with different production dates were bought. Thus, a total of 219 bottles were purchased and transferred to the laboratory of for determination of methanol concentration. 

The production and expiration date of all samples were controlled and recorded. The production dates of the herbal waters varied between 15 to 60 days prior to the study. 

Gas chromatography (GC) was used for determination of methanol concentration. The GC instrument used was Varian CP-3800 () chromatograph equipped with a fused capillary column coated with silica CP-sil5CB (length: 30 m, inside diameter: 0.25 mm, outside diameter: 0.39 mm, film thickness: 0.10 µm) and flame ionization detector (FID). Injections were made in split mode (split ratio 20:1). Injection port temperature was 170 °C and the oven was programmed from 40 to 210 °C at a rate of 20 °C/min. The FID temperature was 280 °C. The carrier gas was hydrogen with a flow rate of 30 ml/min.

At first the standard methanol curve was prepared in 20, 50, 100, 200, 500 and 1000 mg concentrations. Chloroform, acetone, isopropyl alcohol and benzene were separately used as a candidate for the internal standard. Finally, benzene with a concentration of 1000 mg/l showed the best result in curve type, time out from the device and reproducibility. To each of the methanol concentrations of 20, 50, 100, 200, 500 and 1000 mg/l, 1000 mg/l benzene was added and calibrated. Ten ml of each herbal water was then transferred to a clean pipe, the internal standard was added and then it was injected into the device. 

**Figure 1.**
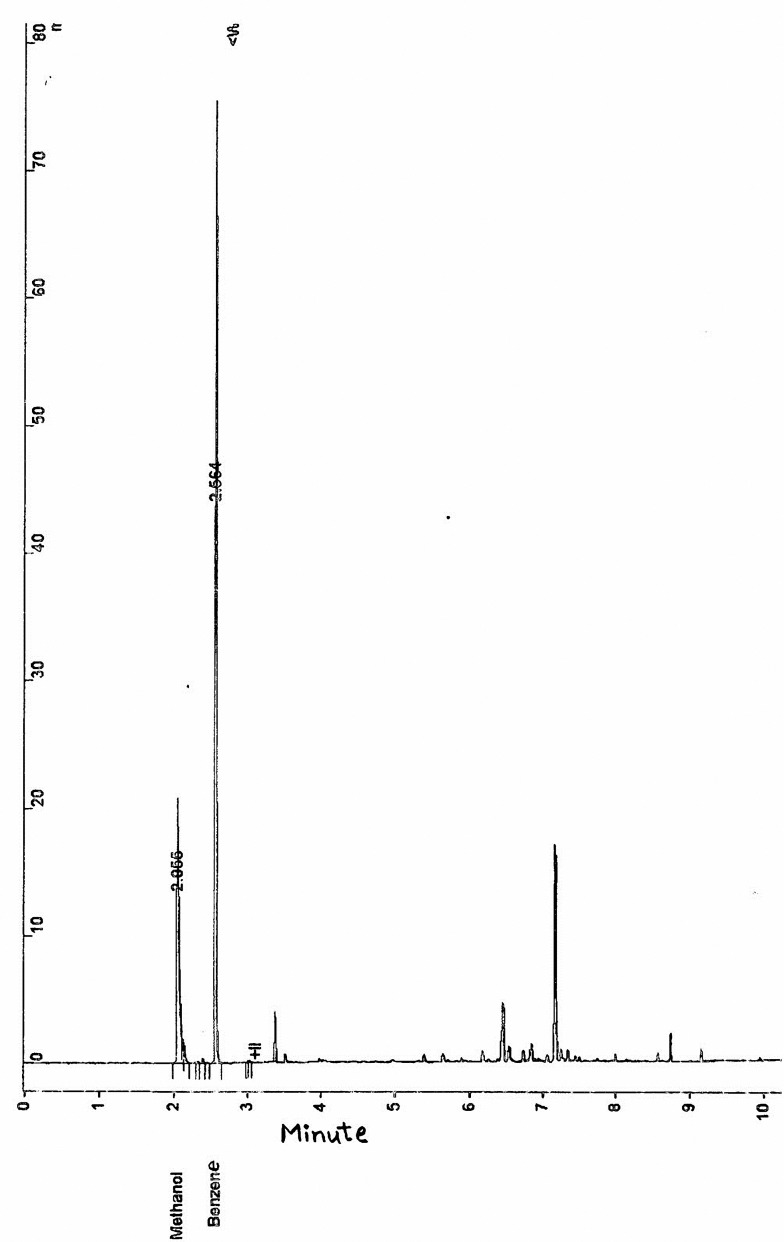
Gas chromatographic peaks of methanol and benzene as the internal standard, obtained from a sample of Cumin herbal water contained 672.7 mg/l methanol.

Three measurements were performed for each sample and the mean was considered as the reported value. Detection limit of methanol concentration was 10 mg/l. Minimum level of quantification was 20 mg/l. Accuracy, precision and reproducibility of the method were 97%, 96% and 98%, respectively. A typical GC peaks of methanol and benzene obtained from a sample of Cumin herbal water containing 672.7 mg/l is shown in Figure 1.

Descriptive statistical analyses including Kruskal-Wallis and Mann-Withny tests were applied using Statistical Package for Social Sciences (SPSS, , version 11.5). A *P*-value≤ 0.05 was considered as the significant level.

## Results

**Figure 2.**
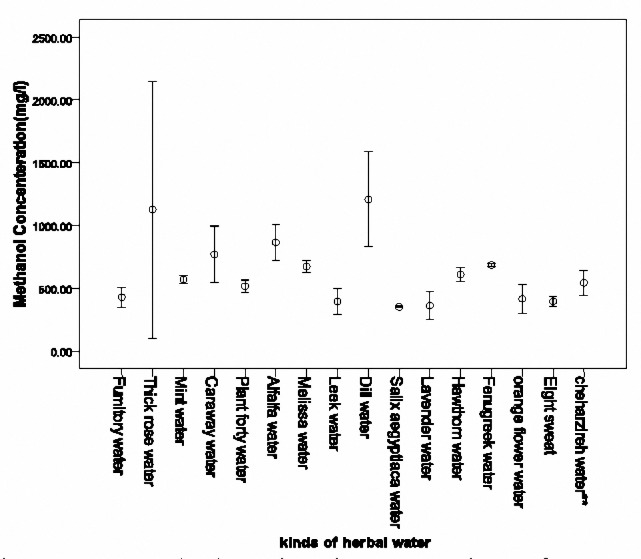
Mean (SD) methanol concentrations of more than 350 mg/l in 16 herbal waters of factory A (350 mg/l was chosen as a cut off to divide the herbal waters into two figures).

Methanol concentration of 38 herbal waters of factory A with a cut off of 350 mg/l divided into two groups. The first group consisted of 16 different herbal waters with methanol concentration of more than 350 mg/l which is shown in Figure 2. The second group included 22 different herbal waters with methanol concentration of 350 mg/l and less, which is shown in Figure 3-1 and 3-2. The highest concentration was detected in Thin rose water (1571.35 mg/l), Dill water (1310.65 mg/l), Alfalfa water (918.33 mg/l) and Mint water (856.25 mg/l), respectively. When considering the mean concentration in the three samples for each herbal water, the highest concentration was measured in Dill water (1208±202.74 mg/l), Thin Rose water (1017.41±59.68 mg/l), Alfalfa water (865.05±80.52 mg/l) and caraway water (769.19±91.18 mg/l), respectively.

**Figure 3-1.**
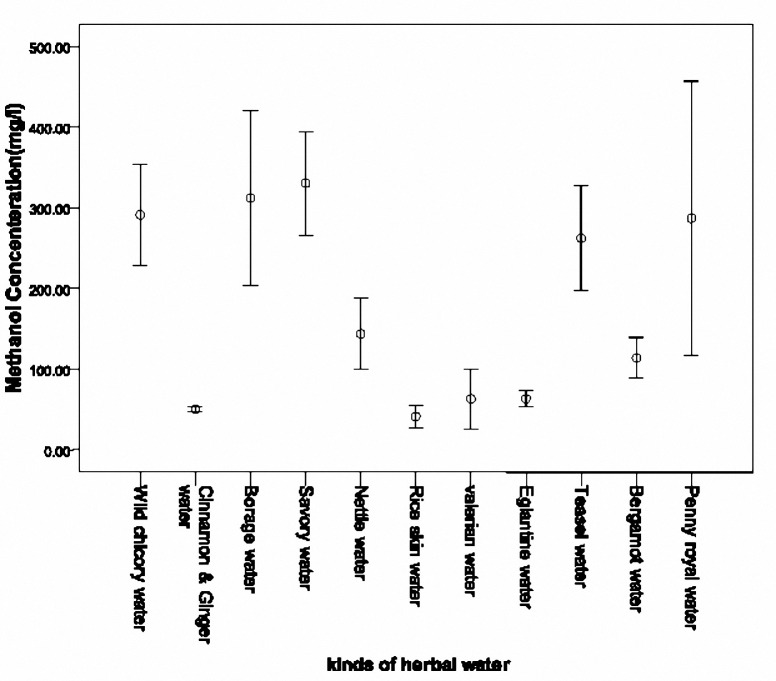
Mean (SD) methanol concentrations of less than 350 mg/l in 11 herbal waters of factory A.

**Figure 3-2.**
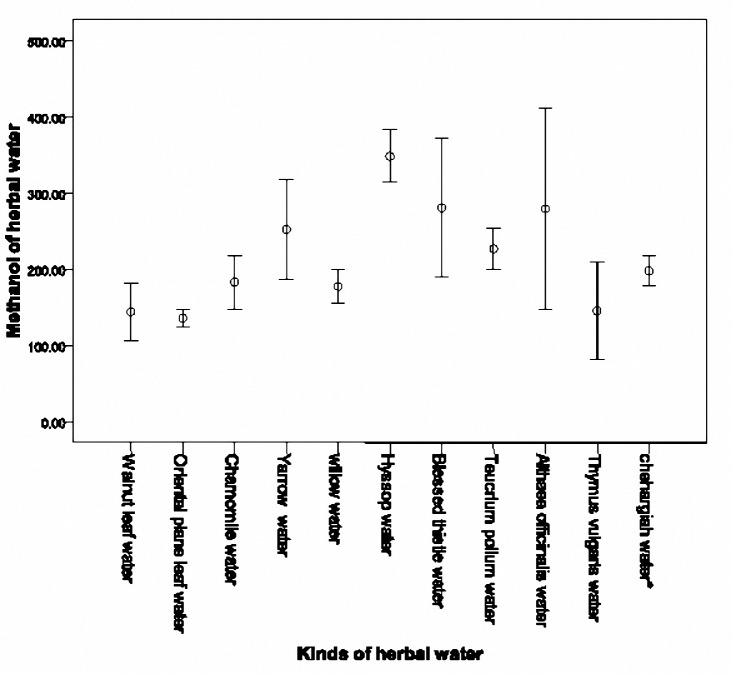
Mean (SD) methanol concentrations of less than 350 mg/l in 11 herbal waters of factory A.

**Figure 4-1.**
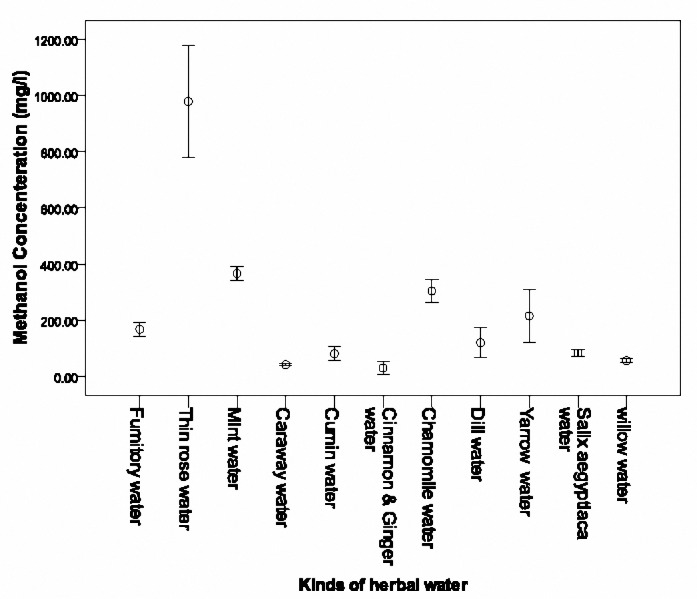
Mean (SD) methanol concentrations of 11 herbal waters of factory B.

**Figure 4-2.**
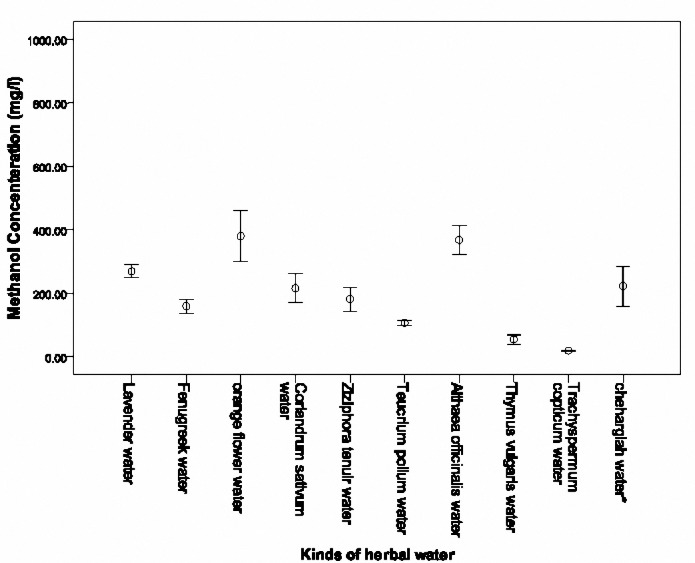
Mean (SD) methanol concentrations of 10 herbal waters of factory B.

**Figure 5.**
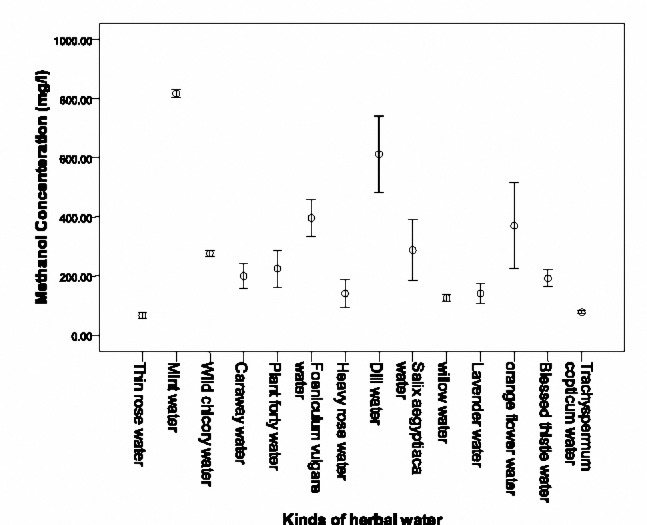
Mean (SD) methanol concentrations of 14 herbal waters of factory C.

Lowest methanol concentration was recorded in Rice Skin water (41.33±7.85 mg/l), Cinnamon and Ginger water (50.12±1.62 mg/l), Valerian water (63.05±20.9 mg/l) and Eglantine water (63.20±5.81 mg/l), respectively.

Methanol concentration in the 21 herbal waters of factory B is shown in Figure 4-1 and Figure 4-2. The highest methanol concentration was detected in Thin rose water (978.52±92.81 mg/l), Mint water (366.13±9.41 mg/l) and flower water (380.25±42.26 mg/l), respectively. Lowest methanol concentration was observed in Trachyspermum copticum water (18.93±1.04 mg/l), Cinnamon and Ginger water (29.64±10.88 mg/l), Cumin water (41.37±1.17 mg/l), respectively.

Methanol concentration in the 14 herbal waters of factory C is demonstrated in Figure 5. Highest concentration was in Mint water (817.47±7.80 mg/l), Dill water (611.93±70.76 mg/l) and Cumin water (396.19±29.00 mg/l).

Lowest methanol concentration was revealed in Thin rose water (66.73±5.68 mg/l), Trachyspermum copficum water (77.69±3.20 mg/l) and Willow water (125.2±5.71 mg/l).

Comparison of methanol concentration in herbal waters commonly produced in all the three factories has been illustrated in Table 1. Rose water methanol concentrations in the factories of A and B were much higher (*P*= 0.003) than in the factory C. Whereas in Mint waters, were in reverse. In summary, apart from Orange flower water, methanol concentrations of the other herbal waters were significantly different between the factories (Table 1).

Comparison of methanol concentration in the herbal waters commonly produced in factories A and B is shown in  Table 2. Methanol concentration in Matricaria chamomilla and Althaea officinalis water of factory B was higher than A, whereas in all the other produced herbal waters, factory A stood before factory B in term of methanol concentration. Mean methanol concentrations in herbal waters of factories A, B and C were 374.69 mg/l, 209.81 mg/l and 280.12 mg/l, respectively in which a statistically significant difference was observed between factories A and B (*P*< 0.001).

**Table 1.** Comparison of methanol concentration in the herbal waters of the three factories.

	Factories Name			
Herbal Water Name	A	B	C	*P* value
Rose water	1017.41 ± 59.68	978.52 ± 92.81	66.73 ± 5.68	0.003
Mint water	568.03 ± 12.81	366.13 ± 9.41	817.47 ± 7.80	<0.0001
water	177.53 ± 11.01	55.56 ± 3.21	125.20 ± 5.71	<0.0001
Lavender water	362.31 ± 52.76	269.50 ± 9.52	140.97 ± 15.84	0.01
Orange flower water	414.74 ± 54.56	380.25 ± 42.26	370.51 ± 69.08	0.521
Salix aegyptiaca water	351.28 ± 4.41	82.92 ± 5.36	287.46 ± 48.07	<0.0001
Dill water	1208.52 ± 202.74	119.63 ± 24.79	611.93 ± 70.67	<0.0001

**Table 2.** Comparison of methanol concentration in herbal waters of the factories A and B.

	Factories Name		
Herbal Water Name	A	B	*P* value
Fumitory water	425.65 ± 35.10	167.44 ± 11.88	<0.0001
Thymus vulgaris water	145 ± 57.30	55.43 ± 2.64	0.02
Teucrium polium water	226.63 ± 13.44	107.26 ± 3.74	0.002
Fenugreek water	685.66 ± 6.03	159.48 ± 10.28	<0.0001
Matricaria chamomilla water	182.85 ± 19.87	303.67 ± 20.01	0.01
Althaea officinialis water	278.98 ± 75.24	368.22 ± 25.36	0.05

## Discussion

Iranian medicinal plants have long been consumed in Iran, mainly due to their medical and pharmaceutical properties (9-11).

The manufacture of herbal waters is growing every year in significant amounts by factories, workshops, private gardens and houses and the products are widely marketed. Application of such products, unlike some other countries is quite common in . On the other hand, due to differences in terms of production, consumption and cultural issues, comparing our studies with those conducted on substances such as distilled alcohol in other countries will be hardly possible. It is important to note that numerous reports have proven the pollution to methanol and other harmful compounds like heavy metals in alcoholic beverages and even fruit and vegetable juices which clearly shows the importance of this field for further research (12,13).

It seems that methanol is induced during the production or storage of such herbal waters via the effects of enzymes on pectins in the cell wall. One of these important enzymes is pectin methylesterase-1 (PME) which can demethylise pectin and release methanol (14,15). Therefore, it could be concluded that the existence of more wooden organs in a plant could make it more prone to methanol production.

In a study, it has been shown that the soaking time increase methanol production (16). Industrial compression in comparison to the manual method has also been proved to increase the amount of released cell wall enzymatic materials. Pasteurized herbal waters had a lower concentration of methanol when compared to fresh ones, because the pasteurization process reduces the enzyme activity (4). According to these findings it can be stated that in producing herbal waters if the wooden structure of a plant is omitted to the highest possibility and direct steam distillation is used during production- causing lower enzyme activity and thus methanol concentration will be much less in the end-product.

Application of gas chromatography (GC) in determination of methanol concentration was first developed by Caggiano and Beck in 1963. Since then methanol concentrations were measured in spirits. The averages of methanol content of white wines from different countries were reported as follows: French wines 60 mg/l, Italian wines 60 mg/l, Portuguese wines 63 mg/l, Turkish wines 58.5 mg/l. According to the International Office of Vine and Wine (OIV), the maximum acceptable level for methanol in white wines is 150 mg/l. The averages of methanol content of red wines from different countries were also reported as follows: French wines 163 mg/l, Italian wines 103 mg/l, Spanish wines 145 mg/l, Portuguese wines 195 mg/l, Turkish wines 113 mg/l. According to OIV, the maximum acceptable level for methanol in red wines is 300 mg/l (16-18).

Different types of edibles including fruits, vegetables, fermented beverages, and foods sweetened with aspartame, which breaks down to methanol in the gastrointestinal tract may be ingested with Low doses of methanol. For the above reasons, certain low levels are now accepted for methanol concentration in various foods (19).

The study of Karimi and his colleagues (20) accomplished in 2007 on 10 different types of herbal waters (done by spectrophotometry) reported the highest amount of  Methanol in Dill water (1447/7±23/8 mg/l) and the lowest in Salix aegyptiaca water (79/4±3 mg/l) (20). Because the six investigated factories were not named in the mentioned study, the reported results can not be compared with our results. However, in the current study methanol concentration of Dill water of factory "A"(1208/52±202/7 mg/l) and factory "C" (611/93±70/7 mg/l) were also high whereas the same value was low in Salix aegyptiaca water of factory "B" (82/92±5/36 mg/l).

In the study by Solhi *et al* (21) conducted in 2009 on 6 different types of herbal waters (done by spectrophotometry), the highest amount of methanol was reported in mint water (415.04 mg/l) and the lowest in Fenugreek water (60.26 mg/l). In our study, methanol concentration of mint water of factory "A"(568.03±12.81 mg/l) and factory "C" (817.47±7.80 mg/l) were also high, whereas it was low in Fenugreek water of factory "B" (159.48±10.28 mg/l).

Considering the fact that methanol was detected in all studied samples and due to its high toxicity, regular consumption of these herbal waters, particularly those with high concentration can cause major complications. It is thus required that all processing steps from sorting of herbs or plants to the distillation to be improved to reduce methanol concentration. It is also recommended to perform a toxicology evaluation to ensure that methanol and other possible toxic substances in the end-products are less than the maximum allowable concentrations. Since maximum allowable concentrations for herbal waters in have not been formally established, the health ministry and the standard organization should work on this subject and finalize regulations and control the safety of herbal waters before releasing the products into the market.

This study was aimed on measuring methanol concentration solely in herbal waters. Estimation of methanol in other beverages and other possible toxic compounds such as other alcohols, aldehydes, ketones, nitrates, nitrites and heavy metals in herbal waters is recommended.

## Conclusion

1. Methanol concentration in all herbal waters especially rose water of the three producers was very high that may induce toxicity in people taking these products regularly for a long time. 

2. Improvement of the production process of herbal waters in the factories is required to reduce methanol and other possible toxic compounds.

3. The health department should control the safety of herbal waters before releasing to the market.

4. Standardization of herbal waters is also recommended.

## References

[1] Chuwers P, Osterloh J, Kelly T, Alessandro A, Quinlan P, Becker C (1995). Neurobehavioral effects of low-level methanol vapor exposure in healthy human volunteers. Environ Res.

[2] Batterman SA, Franzblau A, Arcy JB, Sargent NE, Gross KB (1998). Breath, urine, and blood measurements as biological exposure indices of short-term inhalation exposure to methanol. Int Arch Occup Environ Health.

[3] Wang ML, Wang JT, Choong YM (2004). A rapid and accurate method for determination of methanol in alcoholic beverage by direct injection capillary gas chromatography. J Food Compost Analysis.

[4] Bouchard M, Droz PO, Carrier G (2001). A iologically based dynamic model for predicting the disposition of methanol and its metabolites in animals and humans. Toxicol Sci.

[5] Lee CY, Smith NL, Nelson RR (1979). Relationship between pectin methylesterase activity and the formation of methanol in concord grape juice and wine. Food Chem.

[6] Micheli F (2001). Pectin methylesterases: cell wall enzymes with important roles in plant physiology. Trends Plant Sci.

[7] Kuo CC, Wen YH, Huang CM, Wu HL, Wu SS (2002). A removable derivatization HPLC for analysis of methanol in Chinese liquor medicine. J Food Drug Anaylsis.

[8] Zocca F, Lomolino G, Curioni A, Spettoli P, Lante A (2007). Detection of pectinmethylesterase activity in presence of methanol during grape pomace storage. Food Chem.

[9] Karimi Gh, Khoei A, Omidi A, Kalantari M, Babaei J, Taghiabadi E (2010). Protective effect of aqueous and ethanolic extracts of portulaca oleracea against cisplatin induced nephrotoxicity. Iran J Basic Med Sci.

[10] Gholamhoseinian A, Fallah H, Sharifi-far F, Mirtajaddini M (2008). The inhibitory effect of some Iranian plants extracts on the Alpha Glucosidase. Iran J Basic Med Sci.

[11] Hosseinzadeh H, Sadeghnia HR, Imenshahidi M (2009). Review of the pharmacological and toxicological effects of Salvia leriifolia. Iran J Basic Med Sci.

[12] Paine A, Davan AD (2007). Defining a tolerable concentration of methanol in alcoholic drinks. Hum Exp Toxicol.

[13] Szucs S, Sarvary A, McKee M, Adany R (2005). Could the high level of cirrhosis in Central and Eastern Europe be due partly to the quality of alcohol consumed. Addiction.

[14] Laats MM, Grosdenis F, Recourt K, Voragen AGJ, Wichers HJ (1997). Partial purification and characterization of pectin methylesterase from green beans (Phaseolus Vulgaris L.). J Agric Food Chem.

[15] Anthon GE, Barrett DM (2006). Characterization of the temperature activation of pectin methylesterase in green beans and tomatoes. J Agric Food Chem.

[16] Cabaroglu T (2005). Methanol contents of Turkish varietal wines and effect of processing. Food Control.

[17] Nykanen L, Suomalainen H (1983). Aroma of beer wine and distilled alcoholic beverages.

[18] OIV (1990). Recueil des methods internationals d’analyse des vins et des mouts.

[19] Newsome RD (1993). Sugar substitutes. Low Calorie Foods Handbook.

[20] Karimi Gh, Hasanzadeh M, Shahidi N, Samiei Z (2008). Quantitative determination of methanol in plant water produced in Mashhad by spectrophotometry method. J Med Plants.

[21] Solhi H, Delavar M, Abdollahi M (2009). Comparison of methanol concentration in handmade herbal essences produced in Arak city with industrial produced herbal essences with different commercial brands. J Arak Univ Med Sci.

